# Understanding neural signals of post-decisional performance monitoring: An integrative review

**DOI:** 10.7554/eLife.67556

**Published:** 2021-08-20

**Authors:** Kobe Desender, K Richard Ridderinkhof, Peter R Murphy

**Affiliations:** 1 Brain and Cognition, KU Leuven Leuven Belgium; 2 Department of Neurophysiology and Pathophysiology, University Medical Center Hamburg-Eppendorf Hamburg Germany; 3 Department of Psychology, University of Amsterdam Amsterdam Netherlands; 4 Amsterdam center for Brain and Cognition (ABC), University of Amsterdam Amsterdam Netherlands; 5 Trinity College Institute of Neuroscience, Trinity College Dublin Dublin Ireland; École normale supérieure, PSL University, INSERM France; Radboud University Netherlands

**Keywords:** performance monitoring, evidence accumulation, drift diffusion model, error positivity, cognitive control

## Abstract

Performance monitoring is a key cognitive function, allowing to detect mistakes and adapt future behavior. Post-decisional neural signals have been identified that are sensitive to decision accuracy, decision confidence and subsequent adaptation. Here, we review recent work that supports an understanding of late error/confidence signals in terms of the computational process of post-decisional evidence accumulation. We argue that the error positivity, a positive-going centro-parietal potential measured through scalp electrophysiology, reflects the post-decisional evidence accumulation process itself, which follows a boundary crossing event corresponding to initial decision commitment. This proposal provides a powerful explanation for both the morphological characteristics of the signal and its relation to various expressions of performance monitoring. Moreover, it suggests that the error positivity –a signal with thus far unique properties in cognitive neuroscience – can be leveraged to furnish key new insights into the inputs to, adaptation, and consequences of the post-decisional accumulation process.

## Main text

Many of the choices that we make every day are based on noisy information, generated in environments that are complex and subject to change. In the face of such sources of uncertainty, it can be of vital importance to evaluate the accuracy of these choices. Estimating confidence in our decisions and detecting mistakes even without external feedback allows us to identify when future behavior should be altered; for example, by taking a more cautious approach to future decisions when we think we have just made an error.

How the brain achieves this feat of performance monitoring without feedback has been a fruitful topic of research in recent decades, and related physiological signals have been identified across several measurement modalities. The first of these came in the form of deflections in the human event-related potential (ERP) that occur following responses on various kinds of choice tasks and were found to be sensitive to whether the executed choice was correct or erroneous (Falkenstein et al., 1991; Falkenstein et al., 2000; Gehring et al., 1993). It was later observed that these signals are sensitive not only to error commission, but also error *detection* (or ‘error awareness’; Dhar et al., 2011; Endrass et al., 2007; Nieuwenhuis et al., 2001; O'Connell et al., 2007; Steinhauser and Yeung, 2010; Wessel et al., 2011) – that is, whether a committed error was subsequently recognised as such by the participant – and inferred (Scheffers and Coles, 2000; Shalgi and Deouell, 2012) or self-reported (Boldt and Yeung, 2015) decision confidence. Human neuroimaging studies using similar experimental paradigms have identified brain areas where blood-oxygen-level-dependent (BOLD) activity exhibits similar sensitivities (Carter et al., 1998; Fleming et al., 2018; Harsay et al., 2018; Hester et al., 2005; Klein et al., 2007). In animal models, post-decisional but pre-feedback firing rates of single neurons in regions of frontal cortex have been shown to encode the preceding decision (Tsujimoto et al., 2009), its accuracy (Stuphorn et al., 2000), and the tendency to opt-out following initial decisions (Middlebrooks and Sommer, 2012) or wait for a delayed reward (Kepecs et al., 2008; both proxies for decision confidence).

The rich collection of findings summarized above suggests that the brain is well-endowed with signals that can be exploited for performance monitoring when feedback from the environment is not available. However, much of this work has been primarily descriptive, highlighting relationships of the neural signals in question to conditions (e.g. correct *vs.* error), quantities (e.g. graded confidence judgements) and consequences (e.g. next-trial behavioral adjustment) relevant to monitoring. While some authors have begun to interpret these phenomena and antecedents in mechanistic and sometimes also computational terms (e.g. Fleming and Daw, 2016; Kepecs et al., 2008; Yeung and Summerfield, 2012), our aim is to construe an overarching, mechanistically explicit framework that allows us to capture and predict such processes in a more systematic and unifying fashion.

In the current work, we seek to provide such a framework. We pay particular attention to recent findings pertaining to a slow positive wave ERP with a centro-parietal scalp distribution and a peak typically between 200 and 600 ms after choices. Since a cardinal characteristic of this signal is that its amplitude is larger after errors than correct choices (Falkenstein et al., 1991), it is usually referred to as the error positivity, or Pe. We argue that the Pe reflects the computational process of post-decisional evidence accumulation: a proposal that accounts not only for the classical association of this signal with error detection (Dhar et al., 2011; Endrass et al., 2007; Nieuwenhuis et al., 2001; O'Connell et al., 2007; Steinhauser and Yeung, 2010; Wessel et al., 2011), but also specific aspects of its morphology (Murphy et al., 2015) and more recent links with graded confidence judgments (Boldt and Yeung, 2015) and future behavioral adjustments (e.g. Desender et al., 2019a). In turn, we suggest that the Pe – a signal with thus far unique properties in cognitive neuroscience in that it appears to track the dynamic trajectory of a selective ‘metacognitive’ decision variable – provides the critical link to a computational framework for performance monitoring within which the functional significance of related brain signals can be readily understood.

In order for the reader to fully appreciate the details of our proposal, we will start with a brief overview of evidence accumulation models and associated neural signatures. Then, we will explain how post-decisional evidence accumulation provides a mechanistic framework for understanding the Pe. We will end by outlining novel predictions and future research directions arising from our proposed framework.

## Choices through evidence accumulation

**Figure 1.**
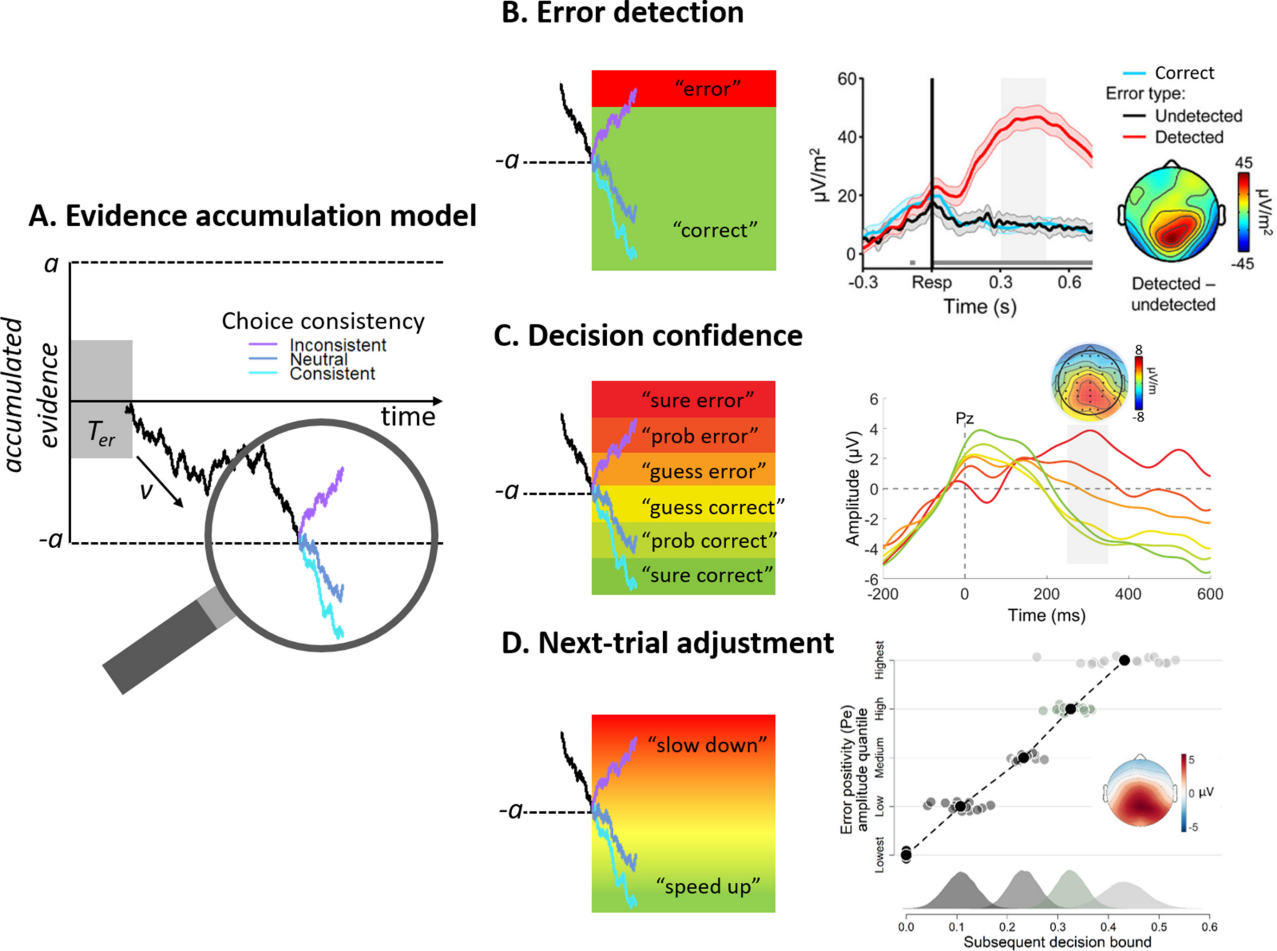
The Pe as a signature of post-decisional evidence accumulation. (**A**) Schematic of pre- and post-decisional evidence accumulation. In the pre-decisional period of a two-alternative forced-choice task, noisy sensory evidence accumulates over time until the decision variable (DV; black trace) reaches one of two bounds (a or -a), corresponding to the two available choice alternatives. After the DV reaches a boundary and triggers a choice, evidence accumulation continues during the post-decisional period (informed by the same sensory evidence and/or other sources of ‘error evidence’). In the depicted example, post-decisional evidence can either further confirm and thus be consistent with the choice made (cyan trace), it can be inconsistent with the choice just made (purple trace) or not really informative about the preceding choice (marine blue trace). In the other panels, we focus on how such post-decisional accumulation can explain different expressions of performance monitoring. (**B**) Error detection. Post-decisional accumulation can produce error detection by assuming that participants impose another bound, represented here by the transition between the green and red area (left panel). Only when the post-decisional accumulated evidence ends up above that additional boundary (i.e. in the red area) will participants indicate that they made an error. Consistent with this, the Pe gradually ramps up preceding the detection of errors, whereas it is diminished in amplitude or even absent for undetected errors (right panel). (**B**) Reproduced from Figure 2 Murphy et al., 2015. (**C**) Decision confidence. Post-decisional accumulation can produce graded confidence judgments, by assuming the accumulated post-decisional evidence is compared against multiple discrete criteria, represented here by the six colored areas (left panel). The indicated level of confidence then directly depends on the category into which the DV falls. Consistent with this, Pe amplitude has been shown to scale inversely with reported level of confidence (right panel). (**C**) Reproduced from Figure 3 Boldt and Yeung, 2015. (**D**) Adaptive modulation of next-trial speed-accuracy tradeoff. The DV furnished by the post-decisional accumulation process can serve as the basis for altering subsequent decision policy. Specifically, depending on the accumulated evidence that a preceding choice was incorrect, the decision boundary can be changed to instantiate a more cautious policy on the subsequent trial and thus decrease the probability of consecutive errors (left panel). Consistent with this, the Pe positively predicts the height of the decision boundary on the following trial (right panel). (**D**) Replotted from Figure 8 Desender et al., 2019a.

Convergent lines of theoretical and empirical work, across psychology and neuroscience, suggest that many kinds of decision can be well explained in terms of the accumulation of evidence for different alternatives over time (Gold and Shadlen, 2007; Ratcliff and McKoon, 2008). According to this framework, in the simplest case of a task with two discrete choice alternatives, an agent is assumed to set two decision boundaries, corresponding to each choice option (see Figure 1A). When presented with noisy evidence (e.g. samples of sensory information in the case of a perceptual choice), the agent then accumulates the evidence for each option, and the option for which the accumulated evidence first transgresses the associated decision boundary is selected, indicating choice commitment. Algorithmic models incorporating these features have been highly successful in fitting choice behavior across many domains (Forstmann et al., 2016; Ratcliff et al., 2016; Shadlen and Kiani, 2013).

A key prediction of models of decision-making through evidence accumulation is that the momentary accumulated evidence – also known as the decision variable (DV) – is represented in the brain. Indeed, signals that appear to track the evolution of the DV have been observed in rodents (Hanks et al., 2014), monkeys (Gold and Shadlen, 2007), and humans (Donner et al., 2009; Heekeren et al., 2004; O'Connell et al., 2012). Of particular relevance here, using scalp electroencephalographic (EEG) recordings, it has been shown that a centro-parietal positivity (CPP) that emerges during choice formation closely resembles a form of the DV (Kelly and O'Connell, 2013; O'Connell et al., 2012; O'Connell et al., 2018). Specifically, in two-alternative decision-making tasks, the CPP bears hallmarks of an *unsigned* (i.e. not choice-selective) DV that can be read out from evidence accumulation models by taking the absolute of the accumulated evidence (Kelly and O'Connell, 2013; Twomey et al., 2016). Three key dynamical properties of this measure are also seen in the CPP on such tasks: (i) a gradual ramping over time, the slope of which depends on absolute evidence strength and correlates with response time (RT); (ii) a stereotyped peak amplitude that is independent of the evidence strength (putatively reflecting a termination of the accumulation process upon boundary crossing); and (iii) a close correspondence between peak latency and RT (putatively reflecting the timing of the boundary crossing). The CPP on these tasks is observed irrespective of the modality of the evidence (e.g. visual vs. auditory) and the method of commitment (e.g. overtly, with a motor response, or covertly without a measurable motor response), suggesting it is a supramodal, effector-independent decision signal.

Another salient characteristic of the CPP manifests in paradigms requiring detection of faint sensory stimuli (as opposed to discrimination of features of high-intensity stimuli). Here, the CPP exhibits the properties described above only on trials when the stimulus is judged to be present (‘hits’ and, to a less clear extent, ‘false alarms’; O'Connell et al., 2012; Twomey et al., 2015). When the stimulus is presented but not detected (‘misses’), however, the signal is diminished in amplitude or even absent entirely. In these respects, as well as in topographic distribution, there are clear similarities between the CPP and the classic P300 component (Hillyard et al., 1971; Squires et al., 1973; Twomey et al., 2015). The diminution of these signals on miss trials can be easily explained in the context of evidence accumulation models as a case in which a ‘stimulus present’ decision boundary remains unmet due to insufficient evidence. By contrast, this family of signals has not been found to ramp up as clearly when participants are asked to actively confirm the absence of the target stimulus (‘correct rejections’; Hillyard et al., 1971; Squires et al., 1973), suggesting the CPP may not reflect accumulation of internally generated ‘evidence for absence’ (Deco et al., 2007) in detection tasks.

In sum, decision-making has been successfully modelled as the accumulation of noisy evidence over time, and a centro-parietal positivity in the EEG has been proposed to reflect key aspects of this process.

## Performance monitoring through post-decisional evidence accumulation

Having described how the process of evidence accumulation provides a plausible account of choice formation in brain and behavior, we now turn to the question of monitoring of choice accuracy in the absence of external feedback. How are decision-makers able to detect their own mistakes without explicit feedback from the environment, and provide fine-grained estimates of confidence in the adequacy of the preceding choice? In order to account for performance monitoring of choices within the same evidence accumulation framework described above, Pleskac and Busemeyer, 2010 proposed that an evidence accumulation process can take place in the post-decisional period and be used to inform estimates of choice confidence (see Figure 1A). To intuit how such a model explains monitoring of choice accuracy, consider a series of choices made with moderately high evidence strength. For the majority of trials, the DV will reach the boundary corresponding to the correct choice, and post-decisional accumulation of samples from the same evidence source will, on average, furnish accumulated evidence that corroborates the selected choice. This corroborating evidence in turn produces a sense of high confidence. For the few incorrect choices, by contrast, post-decisional accumulation will, on average, furnish accumulated evidence that *conflicts* with the selected choice. This conflicting evidence in turn results in reduced confidence, doubt, or even a change of mind (van den Berg et al., 2016; Resulaj et al., 2009). Thus, post-decisional evidence accumulation is a candidate mechanism through which performance monitoring can occur. At this point, it is important to clarify that post-decisional processing is not necessarily restricted to the period following an overt behavioral response, but can in principle also take place before any response is executed. This is because there is usually a small delay between a boundary crossing corresponding to initial decision commitment, and subsequent motor execution – a delay that constitutes one component of the so-called non-decision time parameter *T_er_* in evidence accumulation models (visualized by the gray box in Figure 1A; the other component being stimulus encoding time). It has been shown that if the delay between initial boundary crossing and subsequent response completion is sufficiently long (for example, on tasks requiring response execution via protracted limb movement), then post-decisional accumulation during this delay can produce changes of mind before the initially chosen response is completed (Resulaj et al., 2009).

Because ‘performance monitoring’ is a rather broad concept, let us explore further how the simple mechanism of post-decisional accumulation might account for different expressions of performance monitoring (Yeung and Summerfield, 2012). First, post-decisional accumulation can explain categorical expressions of error detection. For example, in many studies of performance monitoring participants are required to signal (e.g. via button-press) when they think that they made an error with a preceding choice (for detailed discussion, see Wessel, 2012). Such error signalling responses can readily be modelled in the context of post-decisional accumulation by imposing a boundary on the post-decisional process (which is distinct from the boundary corresponding to the initial choice; Figure 1B): Only when the accumulated post-decisional evidence crosses that boundary is an error-signalling response given (Pleskac and Busemeyer, 2010). As alluded to above, the same principle can also be applied to generate overt changes of mind, when an initial choice (corresponding to the initial boundary crossing) is subsequently overturned in favour of a different alternative (corresponding to a secondary boundary crossing; Resulaj et al., 2009). Second, post-decisional accumulation also naturally explains graded levels of confidence in the accuracy of a choice (Figure 1C). Human participants can typically provide well-calibrated estimates of their own performance (Aitchison et al., 2015; Fleming et al., 2018; Sanders et al., 2016). To explain such behavior, it suffices to assume that confidence directly reflects (a transformation of) the accumulated post-decisional evidence. Such an account can explain various patterns that are apparent in empirically observed human confidence judgments (Moran, 2015; Pleskac and Busemeyer, 2010; Yu et al., 2015). For example, it explains why confidence decreases monotonically as difficulty increases (i.e. because post-decisional evidence will, on average, strongly favour the initial, usually correct choice when evidence strength is high), why the accuracy of confidence judgments is better under speed pressure (i.e., because speed pressure increases the probability of ‘fast errors’ even for high evidence strength, which are in turn more likely to generate conflicting evidence in the post-decisional period and be correctly detected; Desender et al., 2021; Desender et al., 2020), and why the accuracy of confidence judgments increases when more time elapses between the first decision and the confidence judgment (Yu et al., 2015). Thus, post-decisional accumulation explains key expressions of performance monitoring (error detection and confidence) and accounts for a range of related findings.

The preceding summary of post-decisional evidence accumulation and its explanatory power vis-à-vis empirically-observed performance monitoring behavior is predicated on the assumption that the post-decisional process operates largely on the same evidence source as the initial choice (Calder-Travis et al., 2020; Pleskac and Busemeyer, 2010; Resulaj et al., 2009; van den Berg et al., 2016). This effective continuation of the initial accumulation is possible in perceptual decision tasks, for example, if the stimulus display remains accessible in the post-decisional period (Fleming et al., 2018), or through accumulation of sensory information that is still in the processing pipeline (e.g. in a sensory buffer) if the stimulus is removed upon choice (Resulaj et al., 2009). Under such a view, the quality of the evidence (or ‘drift rate’) informing the initial and post-decisional accumulation processes will be approximately the same (excluding possible choice-induced biases in evidence weighting; e.g. Rollwage et al., 2020; Talluri et al., 2018; Yu et al., 2015). However, post-decisional accumulation may not be restricted only to the same evidence source that informed the initial choice. Several scenarios are possible where the initial evidence source is no longer available, and other sources of information can be consulted to inform the post-decisional accumulation process. One likely candidate is a short-term memory trace of the stimulus (Smith and Ratcliff, 2009; Vlassova and Pearson, 2013). These traces are known to persist for several seconds after stimulus offset (Wimmer et al., 2015), making them well positioned to furnish input to a deliberative post-decisional accumulation process. Because memory is subject to decay over time, this could produce a case where drift rates can differ between the initial and post-decisional accumulation processes. Post-decisional accumulation could also incorporate new, qualitatively different sources of information that might carry evidence about whether an error was just made. These sources could include an internally generated conflict signal (Yeung et al., 2004), interoceptive signals (Ullsperger et al., 2010), an estimate of elapsed decision time (Desender et al., 2017; Kiani et al., 2014), new information that comes to mind from memory (Nelson and Dunlosky, 1991), or irrelevant signals such as stimulus familiarity (Glenberg et al., 1982) and previous exposure to the same stimulus (Metcalfe and Finn, 2008).

Another important dimension of any post-decisional accumulation process is what we refer to as its reference frame: effectively, what propositions a DV furnished by the accumulation process will serve to arbitrate between. Generally speaking, pre-decisional evidence accumulation will tend to be concerned with mapping accumulated (sensory) evidence to the available choice alternatives (a ‘stimulus/response reference frame’), and it is possible that post-decisional accumulation is simply a continuation of this process. This scenario, which we illustrate schematically in Figure 1, entails that the mapping of the post-decisional DV onto an internal estimate of confidence will depend on the sign of the initial choice, and so would require an additional transformation before the DV can be used to inform explicit confidence or error detection reports. Alternatively, post-decisional accumulation could take the form of an entirely distinct accumulation process, with a different reference frame that specifically pertains to the *accuracy* of the preceding choice (Pouget et al., 2016). In this scenario, evidence samples consistent with the initial choice will always drive the post-decisional DV in one direction, while those inconsistent with the initial choice will drive it in the opposite direction. Such a change in reference frames for the post-decisional process could confer certain advantages. It would ensure that the resulting DV can be directly used to inform confidence and error detection, without requiring a transformation. It would also mean that the post-decisional process could in principle exploit additional evidence sources, such as those that we outline above, that are specifically informative about the accuracy of the preceding choice. This scope for multiplexing of multiple sources of ‘error evidence’ could serve to increase the accuracy of confidence and error detection judgements. Importantly, we show through simulations (Appendix 1 - Appendix 1—figure 1 and 2) that a distinct post-decisional accumulation process with a ‘choice accuracy reference frame’ generates confidence and error detection behavior with equivalent sensitivity, and is subject to the same effects of experimental factors, as a continuation of the initial accumulation process in a stimulus/response reference frame – even when the former is selectively sensitive to evidence samples that conflict with the initial decision (a possible feature of post-decisional accumulation; Yu et al., 2015). Thus, a switch in reference frames, even coupled with a more selective accumulation of the post-decisional evidence, incurs no associated cost in the accuracy of performance monitoring. This question of the reference frame of post-decisional processing is the subject of ongoing interest (Fleming, 2016; Fleming et al., 2018) and one that we will return to later in this review.

## The error positivity as a neural marker of post-decisional accumulation

In light of the above insight that post-decisional accumulation accounts for a wide variety of findings related to performance monitoring, an emerging question is whether a neural signature of this process can be identified. As outlined earlier, the CPP has been proposed to reflect the evolution of the DV (i.e. accumulated evidence) during the process of choice formation (O'Connell et al., 2018). Here, we argue that another ramping centro-parietal ERP, the Pe, reflects a functionally equivalent evidence accumulation process that takes place *after* the choice is made, thus providing a neural readout of *post-decisional* accumulation. In the following, we discuss the evidence in favour of this proposal with a focus on how the morphology of the Pe is consistent with that of a post-decisional DV, and how this neural signal relates to error detection and decision confidence.

### Morphology and functional characteristics of the Pe

In the previous section, we described how the CPP exhibits three key dynamical properties of a DV in bounded evidence accumulation models: gradual ramping with a rate proportional to RT (and absolute evidence strength); a stereotyped pre-response amplitude; and a close correspondence between peak latency and RT. Importantly, models that include post-decisional evidence accumulation predict the same three signatures for the post-decisional DV if it too is subject to a decision boundary – as is expected to be the case for tasks requiring self-initiated error signalling (see also Moran et al., 2015).

**Figure 2.**
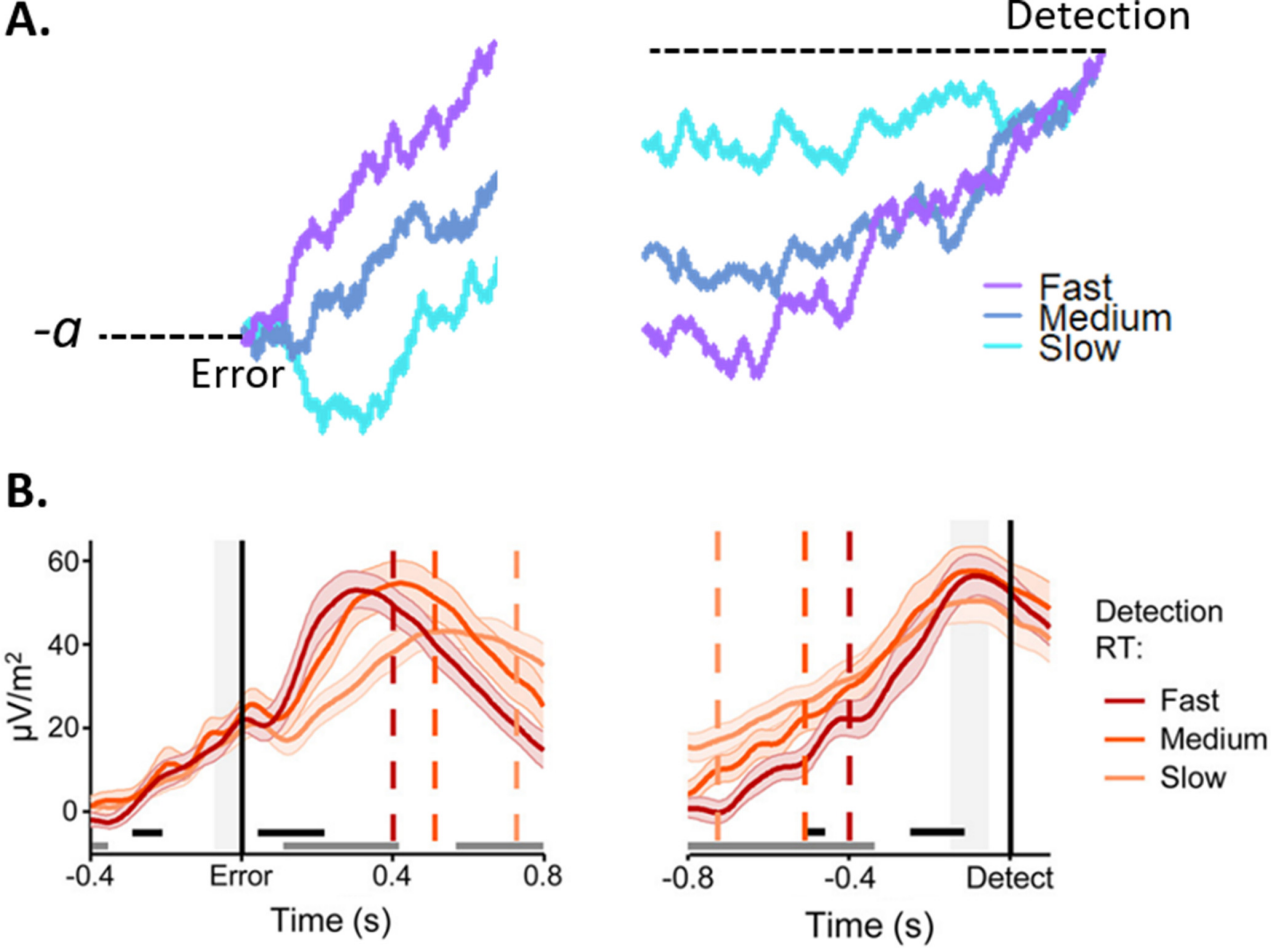
Morphological characteristics of the Pe. (**A**) Schematic of post-decisional evidence accumulation for fast, medium and slow responses when time-locked to error commission (left panel) or to error detection (right panel). (**B**) Average Pe amplitudes separately for fast, medium, and slow detection RTs when time-locked to error commission (left panel) or to error detection (right panel). (**B**) Reproduced from Figure 2 Murphy et al., 2015.

Although the apparent similarity between pre-decisional centro-parietal positivities (the P300 and, by extension, the CPP; Twomey et al., 2015) and the Pe has been appreciated before (Ridderinkhof et al., 2009), it was only recently that the same three morphological signatures described above for the CPP were also observed for the Pe (see Figure 2). In a difficult Go/No-Go task, Murphy et al., 2015 asked participants to signal commission errors by pressing a designated ‘error detection’ button as fast as possible after realizing they had made an error. The three key signatures predicted for a post-decisional evidence accumulation signal were indeed found in the Pe component that was observed between the initial erroneous response and the subsequent error signalling response. First, the build-up rate of the Pe was proportional to the speed of error detection, such that errors that were detected faster were associated with steeper ramping of the Pe (note that here an objective measure of evidence strength was not available, so directly testing an association with evidence strength was not possible). Second, the Pe reached a stereotyped pre-error-response amplitude on detected error trials that was independent of the detection RT. Third, there was a close correspondence between peak Pe latency and the detection RT. Thus, the Pe exhibited all the properties expected of a post-decisional DV in a speeded, categorical error detection scenario. Accordingly, the simulated DV from an algorithmic model of the error detection process driven by evidence accumulation was found to match the trajectory of the Pe remarkably well (Murphy et al., 2015). Aside from these striking morphological similarities, we note that the Pe also shares functional characteristics with the pre-decisional CPP. In the aforementioned study (Murphy et al., 2015), the authors conducted a second experiment where participants did not explicitly signal their errors. A reliable Pe was still observed, indicating that the Pe, like the CPP (O'Connell et al., 2012), is not tied to motor responding. Also like the CPP (O'Connell et al., 2012), the Pe is independent of the sensory modality in which stimuli are presented (Falkenstein et al., 2000).

Importantly, although these three morphological signatures are a first step in relating the Pe to underlying computational principles, such qualitative assessment of these signatures provides a rather indirect approach to do so. A complementary approach to link both aspects would be through model-based EEG analysis, as was previously successfully employed for perceptual decisions (Berberyan et al., 2021; Philiastides and Sajda, 2006; van Vugt et al., 2012) and associated confidence judgments (Pereira et al., 2020). Potential approaches could be to include single-trial Pe amplitudes as regressors when estimating parameters of post-decisional evidence accumulation models (for a related approach, see: Desender et al., 2019b), or to jointly model behavioral data and single-trial Pe amplitudes (Bahg et al., 2020).

Together, these morphological and functional similarities suggest that the CPP and Pe may reflect the same functional process (cf. Ridderinkhof et al., 2009), and thus emphasize the credentials of the Pe as a candidate signature of post-decisional evidence accumulation.

### Error detection and the Pe

Most of the early work on neural signatures of performance monitoring did not place much emphasis on the possible role of the Pe. This was partly because it was unclear whether the Pe truly reflected a distinct neural signal, or was rather produced by the same oscillatory event phase-locked to error commission as the earlier error-related negativity (ERN). This changed with the seminal work by Nieuwenhuis et al., 2001, who were able to dissociate both neural signals. In an anti-saccade task, they asked participants to report their own errors via a manual error signalling response, and they observed a pronounced Pe component for detected versus undetected errors. Contrary to this, the amplitude of the ERN was not modulated by error detection. The association of the Pe with error detection has since been shown to be highly robust (Dhar et al., 2011; Di Gregorio et al., 2018; Endrass et al., 2007; Hughes and Yeung, 2011; Murphy et al., 2012; O'Connell et al., 2007; Wessel et al., 2011), while subsequent studies examining the ERN in this respect have yielded mixed results (see Wessel, 2012).

As alluded to above, the strong association between the Pe and error detection is naturally accounted for by the proposal that the Pe reflects post-decisional evidence accumulation, coupled with the insight that error detection can be treated analogously to a signal detection problem. Within this framework, error detection is determined by the imposition of a boundary on the post-decisional accumulation process. Thus, detected errors (‘hits’, characterized by sufficient accumulated evidence to reach the boundary) will be accompanied by larger Pe amplitudes than undetected errors (‘misses’, where the boundary is not crossed; Figure 1B). According to this interpretation, it should be possible to change the relationship between Pe amplitude and error signalling behavior simply by altering the height of this ‘error detection bound’ (i.e. the transition between red and green in the left panel of Figure 1B). Indeed, Steinhauser and Yeung, 2010 incentivized participants to adopt either a liberal or a conservative criterion to report their errors. As predicted, they observed that Pe amplitudes were larger for signalled errors when participants used a conservative criterion, corresponding to a higher boundary requiring more accumulated evidence to be reached (see also Steinhauser and Yeung, 2012). These findings are again consistent with the view that the Pe reflects post-decisional evidence accumulation and further suggest that, as is the case for pre-decisional accumulation processes (Bogacz et al., 2010; Hanks et al., 2014; Murphy et al., 2016), post-decisional boundaries are under strategic control.

We note here that our framework offers a counterpoint to earlier ideas, informed by the aforementioned findings that Pe amplitude differentiates detected from undetected errors, that this signal might reflect ‘error awareness’ (Nieuwenhuis et al., 2001). In the current framework, the Pe does not reflect awareness of an error per se; rather, it reflects the trajectory of a dynamic evidence gathering process that culminates in commitment to the proposition that an error has just been made.

### Decision confidence and the Pe

Research on the role of the Pe in performance monitoring has been restricted mainly to paradigms like those described above, where participants indicate whether or not they think they made an error (see e.g. Wessel, 2012). However, if error detection results from imposing a single boundary onto this neural signal, it should be possible for participants to provide more fine-grained estimates of the likelihood that a choice was correct by comparing signal amplitude against multiple discrete boundaries (Yeung and Summerfield, 2012). Indeed, Boldt and Yeung, 2015 reported a study in which participants were asked to evaluate their own performance on a graded scale ranging from ‘certainly wrong’ up to ‘certainly correct’. Consistent with our proposal, they found that Pe amplitude varies near-monotonically with the level of confidence that the preceding choice was (in)correct (see Figure 1C): the more certain the participant was that they made an error, the larger the Pe amplitude. Note that this result dissociates the Pe from a pure uncertainty signal, which would exhibit an inverted-U relationship with the scale ratings. Consistent with post-decisional accumulation models described earlier, this study and others Desender et al., 2019b; Selimbeyoglu et al., 2012 have provided evidence that the same process underlies both error detection and decision confidence: they showed that a classifier trained on post-decisional EEG data to dissociate correct responses from errors also generalized to predict fine-grained differences in decision confidence.

The proposal that the Pe encodes decision confidence has not been unequivocally supported by existing data. It is expected that the amplitude of a pure signal of decision confidence should vary monotonically with the reported level of decision confidence. Contrary to this, in the study of Boldt and Yeung, 2015; shown Figure 1B the relationship between confidence and reported Pe amplitude was not perfectly monotonic, instead showing approximately equal signal amplitudes for trials judged as ‘certainly correct’ and trials judged as ‘probably correct’. We note, however, that the temporal window for measurement of the Pe in this study was early (250–350 ms post-response) compared to some other studies of the Pe (e.g. Murphy et al., 2015), and the signal at later time points appeared to exhibit the predicted monotonic relationship with confidence. A reanalysis of these data in a slightly later time window (300–500 ms post-response) did show a monotonic relation between confidence and Pe amplitude, with a significantly different amplitude when comparing ‘certainly correct’ and ‘probably correct’ trials. However, because one should be cautious with a post-hoc analysis in which the time window is not selected a priori (Luck and Gaspelin, 2017), it remains to be seen in future studies whether indeed the Pe, when measured in a slightly later time window, shows a consistent monotonic relation with the reported level of decision confidence.

In the current work, we make the strong point that decision confidence is computed based on the accumulated level of post-decisional error evidence, as reflected by the (post-decisional) Pe component. Importantly, there is some evidence in the literature that confidence computations can already be tracked prior to response execution (Dotan et al., 2018), and it is known that observers are capable of making well-calibrated confidence judgments at the same time as their primary decisions (Kiani et al., 2014). Furthermore, neural signals have been identified that evolve prior to initial decisions that are predictive of later confidence judgments (Gherman and Philiastides, 2018). Nevertheless, we contend that in contexts where probing of confidence is delayed relative to primary decisions, and relevant information might be available in the post-decisional period to inform those judgments (e.g. when access to the stimulus persists after the initial choice, or when the primary response is highly speeded), post-decisional accumulation will play a central role in determining confidence judgments – and furthermore, can improve their accuracy (Yu et al., 2015).

### The Pe reflects accumulation of evidence for an error

One salient aspect of the findings summarized above is that the Pe is generally large in magnitude when a preceding choice is likely to be incorrect (low confidence, or error signalling response), and diminished or entirely absent when the choice is likely to be correct (high confidence, or omission of error signalling response). This insight is likely informative about the nature of the post-decisional process reflected in the Pe in at least two respects. First, it highlights that the Pe is functionally similar to the CPP/P300 in signal detection contexts (Hillyard et al., 1971; O'Connell et al., 2012; Squires et al., 1973; Twomey et al., 2015), and suggests that the post-decisional accumulation process reflected in this signal may be considered analogous to a signal detection problem where the objective is to detect the commission of an error.

Second, this key characteristic of the Pe suggests that it reflects a process that deals specifically in the accumulation of evidence that the preceding choice was incorrect, or ‘error evidence’. At a minimum, this upward ramping of the Pe specifically in response to error evidence would seem to necessitate a reconfiguration of the accumulation process, from a stimulus/response reference frame in the pre-decisional period to a choice accuracy reference frame in the post-decisional period (see above; Fleming et al., 2018; Pouget et al., 2016). In this sense, contrasting with rapid changes of mind that appear to be driven by accumulation of sensory evidence that is still ‘in the pipeline’ at the time of the initial bound crossing (Resulaj et al., 2009; van den Berg et al., 2016), the post-decisional process reflected in the Pe may be considered truly distinct from the pre-decisional accumulation. An important test of this hypothesis, which to our knowledge has not yet been conducted, will be to examine centro-parietal decision signals on a task where the stimulus display remains accessible in the post-decisional period, and participants are instructed to report their confidence in their initial choice after limited post-decisional viewing (Fleming et al., 2018). We predict that while the typical CPP signal evolves during initial decision formation, the post-decisional, pre-confidence judgement signal will only ramp up only on receipt of new evidence that conflicts with the preceding choice.

With this reframing comes the possibility that post-decisional accumulation is informed not only by newly arriving sensory evidence or stimulus information held in short-term memory, but also (or instead) by qualitatively distinct sources of error evidence. Some tentative support for this idea comes from an analysis of the relationship between the Pe and fronto-central theta power – the latter thought to scale with error likelihood, perhaps through encoding of the conflict between competing action plans (Cavanagh and Frank, 2014; Cohen, 2014; Yeung et al., 2004). Single-trial, error-related fronto-central theta power was found to predict successful error detection and to correlate with both detection RT and the build-up rate of the Pe (Murphy et al., 2015) – all properties expected of an error evidence signal providing input to a post-decisional accumulation process that we propose is reflected in the Pe.

## Motivational and functional significance of the Pe

As outlined at the outset of our review, the ultimate goal of performance monitoring is to facilitate adjustments to behavior that are adaptive (Botvinick et al., 2001; Fernandez-Duque et al., 2000; Ridderinkhof et al., 2004; Shimamura, 2008): If a monitoring system detects that several errors have been made in a row, this likely calls for a change in the decision policy or model of the world that produced those errors (Purcell and Kiani, 2016a; Sarafyazd and Jazayeri, 2019). Given the close theoretical link between monitoring and adaptation, we now turn our focus to the possible motivational and functional significance of the Pe and post-decisional accumulation process. Harsay et al., 2012 suggested that *motivational salience* is a useful concept to link performance monitoring and error detection with adaptive behavior. Notable parallels have been observed between both the neural processing and behavioral consequences of errors and other kinds of salient event (such as deviant or novel stimuli; e.g., Notebaert et al., 2009; Ullsperger et al., 2010; Wessel, 2018). Erroneous choices are motivationally significant events because of their usually infrequent occurrence, their usefulness as learning signals, and their utility in triggering behavioral adaptation.

We suggest that the motivational salience of a preceding choice can be read out from the DV of a post-decisional accumulation process that accumulates evidence that that choice was incorrect – that is, precisely the process we propose to be reflected in the Pe. In other words, the accumulation process captured by the Pe furnishes a scalar quantity that can be directly leveraged to determine the desired magnitude of adjustments to future behavior. One possible course of adaptive action is to employ a more cautious decision policy after an error, instantiated by raising the decision boundary on the following trial (Dutilh et al., 2012; Purcell and Kiani, 2016b). This boundary raising will lead to slower decisions and, critically, will also increase the probability of being correct. According to our proposal, the magnitude of such *post-error slowing* (Rabbitt, 1966) should be predicted by the magnitude of the post-decisional DV on the preceding trial (possibly mixed with residual ‘orienting’ effects to the preceding error; Notebaert et al., 2009), which can be read out via the amplitude of the Pe. Consistent with this notion, some early studies showed a relation between Pe amplitude and post-error slowing through between-subject correlations (Hajcak et al., 2003; Nieuwenhuis et al., 2001). However, according to our framework such adaptive slowing should also manifest at the trial-by-trial level. Moreover, it should not be restricted to the categorical contrast of errors vs corrects, but rather should depend on the amount of accumulated post-decisional error evidence (i.e. Pe amplitude) in a graded manner. Consistent with this, Desender et al., 2019a, recently showed that Pe amplitude strongly and linearly predicts the trade-off between speed and accuracy on the next trial (see Figure 1D). These findings are in line with our proposal that the Pe furnishes a scalar quantity that can be adaptively used to alter subsequent decision policy.

While post-error slowing is perhaps the most commonly investigated form of adaptive behavior, other forms can be observed as well and these too may be determined by the outcome of a post-decisional accumulation process. For instance, post-decisional evidence accumulation might also underlie the decision to transition from an exploitative to an explorative mode, or vice versa, in foraging tasks (e.g. Kolling et al., 2012). Although such tasks usually involve veridical feedback about the reward that was just gained, thereby obviating the need for internal computations of choice accuracy, there is some evidence that confidence about the upcoming choice being the most rewarding modulates the trade-off between exploitation and exploration (Boldt et al., 2019). As another example, Desender et al., 2019b showed that Pe amplitude after an initial choice could be used to predict whether or not participants chose to sample more information from a noisy sensory evidence source before confirming or changing that choice. Thus, presumably via simple comparison to a boundary, the post-decisional DV reflected in the Pe can be used to guide choices about whether or not to seek additional information.

## Open issues and directions of future enquiry

We have argued that the Pe reflects post-decisional evidence accumulation. Our proposal not only reconciles a variety of previous research findings; it also opens up exciting avenues of future research. In the remainder of this review, we discuss how our proposal relates to hierarchical accounts of performance monitoring and ‘metacognition’, possible neural origins of the Pe, and the implications of our proposal for the onset of awareness.

### Post-decisional evidence-accumulation, second-order frameworks, and the source of error evidence

According to our proposal, performance monitoring arises from the same computational process that is responsible for the choice itself: the accumulation of evidence. In the literature on performance monitoring, other proposals have characterized metacognitive processes as ‘second-order’ decisions whereby a metacognitive monitor reads out the evidence from a ‘first-order’ decision system that deals with the primary choice (Bang et al., 2019; Fleming and Daw, 2016; Maniscalco and Lau, 2012; Pasquali et al., 2010). Empirical support for such a hierarchical structure of performance monitoring is that some manipulations selectively affect confidence judgments while leaving choice accuracy unaltered (Bang et al., 2019; Boldt et al., 2017; Koizumi et al., 2015; Maniscalco et al., 2016; Peters et al., 2017; Wokke et al., 2017; Zylberberg et al., 2012).

A detailed comparison of the current proposal with such hierarchical models is beyond the scope of this review, although we wish to emphasize that the existence of manipulations that selectively affect confidence is not inconsistent with our framework. Rather, such findings touch directly upon our earlier discussion of the various evidence sources that could be exploited by the post-decisional accumulation process. As noted above, one possible source of input is sensory evidence that is still in the processing pipeline at the time of choice initiation (Resulaj et al., 2009; van den Berg et al., 2016). However, such influences are expected to be rapid and transient, lasting for only the duration of transmission delays from sensory to decision circuits. Indeed, the CPP for initial decisions has been observed to peak significantly *later* than the onset of electromyographic (EMG) activity in the response-executing effector (Steinemann et al., 2018), which may reflect the influence of such residual ‘in-the-pipeline’ evidence. The Pe, by contrast, peaks several hundred milliseconds later still, and so is better positioned to incorporate other inputs such as conflict/error likelihood signals generated in medial frontal cortex around the time of initial choice (Fleming, 2016; Murphy et al., 2015). A significant open question is what factors determine the inputs that drive post-decisional accumulation processes in different contexts. Moreover, the supramodal character of the CPP/Pe family of signals suggests the intriguing possibility that the post-decisional accumulation process might be capable of multiplexing different sources of error evidence. If this is the case, how does the brain assign weights to the different evidence sources?

We hope that the framework proposed here will lay the foundation for new light to be shed on such questions. The Pe as a readout of the post-decisional accumulation process holds significant promise for delineating what computational quantities and associated brain signals might provide evidence for an error in a particular context – whether this be, for example, conflict signals generated in medial frontal cortex in challenging choice RT tasks (Murphy et al., 2015), or information held in hippocampal long-term memory circuits in more deliberative contexts (Nelson and Dunlosky, 1991). Linking variation in candidate neural signals with aspects of Pe morphology that are expected to scale with evidence strength (build-up rate, peak latency) is a clear first step. We believe that such pursuits will greatly illuminate the functional significance of the gamut of neural signals linked to performance monitoring at the outset of this article.

### Neural origins of the Pe

As reviewed above, the Pe component is well characterized in terms of its morphology and relation to error detection and decision confidence. At the same time, the sources of this neural signal remain underspecified. Studies on error detection have often pointed toward an important role for the anterior insula (Ullsperger et al., 2010). For example, Harsay et al., 2018 observed that, while all errors were registered within the rostral cingulate zone, behavioral adaptation followed only when error processing also entailed engagement of the anterior insula, which then recruited autonomic arousal and balanced the activation and connectivity of task-positive networks vis-à-vis the default mode network. However, convincing evidence directly relating the Pe to a neural source is currently lacking. An important reason for this is that accurate source localization is difficult for broadly distributed signals of this nature, and the few studies that have attempted this for the Pe have suggested a quite diverse set of sources (e.g. anterior insula, Dhar et al., 2011; rostral ACC, Herrmann et al., 2004; posterior-cingulate/precuneus, O'Connell et al., 2007). Moreover, the Pe is remarkable in that a functionally equivalent signal has yet to be identified through invasive, single-unit electrophysiology. Indeed, choice-selective neurons that show ramp-like activity during initial decision formation tend to rapidly decrease in firing rate immediately after the decision-reporting action (Gold and Shadlen, 2007). This not only suggests that an exciting future direction lies in identifying the single-unit equivalents of the Pe, which is a vital step in unravelling how post-decisional accumulation is implemented in the brain. But it also highlights how, at least currently, the Pe should hold unique appeal for cognitive neuroscientists interested in performance monitoring and adaptive behavior. A study combining EEG and fMRI to further unravel the functional neuroanatomy of the Pe in human subjects would hold promise (Gherman and Philiastides, 2018; Pereira et al., 2020), as such an approach has already proven useful for identifying likely neural generators of the error-related negativity (Debener et al., 2005; Iannaccone et al., 2015). Such a study would also be able to shed light on the level of overlap in the neural sources generating the CPP and the Pe. This would be highly informative, not least with respect to the question of whether the Pe reflects a continuation of the initial evidence accumulation process in the same neural circuit, or whether it reflects a fundamentally distinct signal.

### The onset of awareness

In this review, we proposed that the Pe reflects a process of post-decisional evidence accumulation that gives rise to metacognitive experiences such as error detection and confidence. This raises the intriguing question of whether processes such as error detection and confidence cannot take place before the peak latency of the Pe (see Di Gregorio et al., 2020). Of particular relevance here is that participants can provide meaningful confidence estimates when providing both choice and confidence simultaneously in a single response (Kiani et al., 2014; Zylberberg et al., 2016). This seems to suggest that confidence computations do not necessarily entirely take place post-decision. In more recent modelling work, however, it was shown that in such designs, qualitative signatures of confidence (such as an interaction between evidence strength and choice correctness; Sanders et al., 2016) can only be captured by an evidence accumulation framework when assuming a slight temporal delay between choices and confidence (Desender et al., 2021). Further work is needed to unravel the precise dynamics involved when reporting both choice and confidence simultaneously, and to shed light on the role of the Pe in this process.

Some recent work identified a neural signature of confidence occurring prior to both the choice and confidence report (Gherman and Philiastides, 2015, Gherman and Philiastides, 2018). However, findings such as these should be interpreted with caution, because pre-choice neural signals that dissociate high from low confidence do not imply pre-choice awareness. For example, it is known that errors can be reliably predicted from alpha band activity tens of seconds before the decision actually takes place (O'Connell et al., 2009). Part of the difficulty in assessing the onset of awareness seems to stem from reliance on paradigms in which participants first make a forced choice about a stimulus, and subsequently indicate their level of confidence in their decision. Recent advances in finger tracking might allow new light to be cast on the question of whether metacognitive experiences such as error awareness and confidence can emerge prior to choice commitment (Dotan et al., 2018; Dotan et al., 2019).

## Limitations

### Evidence accumulation models

The current review leverages evidence accumulation models to explain different expressions of performance monitoring. Therefore, potential limitations of these models should be considered when evaluating the current proposal. A common criticism of ‘vanilla’ evidence accumulation models (the DDM being the most popular example) is that they fail to reproduce certain behavioral effects – for example, the effects of fine-grained manipulations of speed/accuracy trade-off on the precise shapes of correct and error RT distributions (Rafiei and Rahnev, 2021). Another criticism is that certain classes of evidence accumulation model (e.g. those like the DDM that prescribe the diffusion of a single, one-dimensional decision variable) lack biological plausibility, and that alternative formulations (e.g. those prescribing a race between two or more distinct accumulators) are better candidates for realization in neural circuits. To the extent that such criticisms challenge evidence accumulation as the fundamental algorithmic basis for decision-making, they also pose a serious challenge for our account. However, we note in response that augmenting evidence accumulation models with additional features (ideally those supported by neurophysiological findings) has in several cases allowed these models to capture previously problematic features of behavioral data – one example being evidence-independent urgency signals in the case of the speed/accuracy trade-off (Hanks et al., 2014; Murphy et al., 2016; Steinemann et al., 2018). Moreover, one-dimensional diffusion processes like those prescribed by the DDM can be recast as competitive interactions between discrete accumulators (Bogacz et al., 2006; Usher and McClelland, 2001) which in turn can be well approximated by biologically plausible cortical circuit models (Murphy et al., 2021; Wang, 2002; Wimmer et al., 2015). On current balance, we view evidence accumulation as the dominant algorithmic account of how decisions are formed, which in turn provides a solid foundation for the proposal that post-decisional accumulation is responsible for the various aspects of performance monitoring that have been the focus here’.

### A metacognitive decision variable

At the onset of this review, we argued that the Pe might track a selective ‘metacognitive’ decision variable reflecting post-decisional evidence accumulation. Although this idea is appealing, it is worthwhile to acknowledge that it can be challenged on at least two grounds. First, some doubt has been cast on whether a selective metacognitive DV is actually represented in the brain. For the initial decision process itself, it seems well accepted that the brain should have some internal representation of a DV (Gold and Shadlen, 2007; O'Connell et al., 2012). However, this is less clear for performance monitoring. For example, it has been argued that decision confidence can be explained without resorting to ‘metacognitive’ representations (Kepecs et al., 2008; Kiani and Shadlen, 2009). Second, even if such a DV is used by the brain, it should not be taken for granted that an ERP will provide a pure readout of it. It is well understood that ERPs can reflect the superimposition of different neurocognitive processes (Luck, 2014), and it is therefore possible that the Pe should not be interpreted in as straightforward a fashion as our proposal suggests. In this regard, it is imperative to design experimental paradigms that minimize the possibility of mixing of the Pe with other signals and coincident cognitive processes, an approach that was also central to recent studies identifying the CPP with perceptual decision formation (O'Connell et al., 2012). It will also be highly informative to build on the work reviewed above (Steinhauser and Yeung, 2010, Steinhauser and Yeung, 2012) to further examine whether manipulations that selectively influence post-decisional evidence accumulation affect the Pe component in specific ways predicted by our account.

### Conclusion

In the current review, we have discussed the idea that neural signals of performance monitoring can be understood within the context of post-decisional evidence accumulation. Such an account can explain how error detection and decision confidence arise from the same algorithmic process and relate to adaptive behavior. Moreover, it opens up exciting avenues of research concerning the neural origin of post-decisional performance monitoring signals and how these neural signals integrate different sources of evidence for an error.

## Figures and Tables

**Appendix 1—figure 1.**
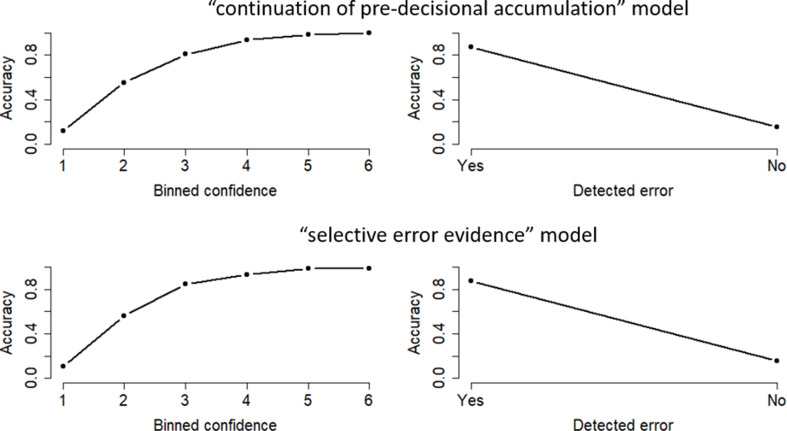
The relation between confidence and accuracy and between error detection and accuracy for the model in which post-decisional accumulation was a continuation of pre-decisional accumulation (top panel) and for the model that selectively accumulates evidence that conflicted with the initial choice (bottom panel).

**Appendix 1—figure 2.**
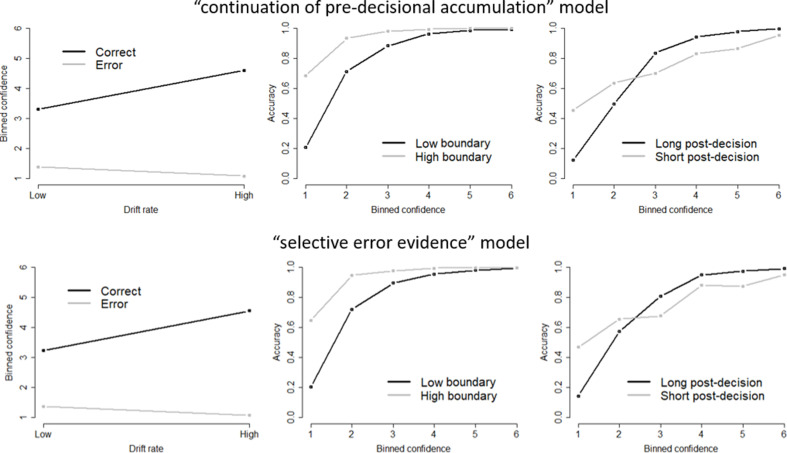
Both models provide qualitatively similar predictions concerning three key findings concerning confidence.

## Appendix 1

### Post-decision accumulation as a continuation of pre-decisional accumulation vs selective accumulation of error evidence

In the simulations reported below we demonstrate that the same behavioral expressions (i.e. error detection responses and confidence reports) can be generated, with comparable accuracy, when modeling post-decisional accumulation as a continuation of pre-decisional accumulation or when modeling it as a distinct post-decisional process driven only by error evidence – that is, only by evidence samples that conflict with the initial decision. In both cases, the magnitude of the decision variable (DV) after post-decisional accumulation is used to inform discrete error detection (via imposition of a single criterion) or graded confidence (via comparison against multiple criteria) judgments. For both models, we simulated 5000 trials of a bounded accumulation model with an unbiased starting point, with drift rate, decision boundary and within-trial noise all fixed to one and non-decision time fixed to 0.2. After boundary crossing, post-decisional accumulation took place for a fixed duration of 1 s. The code for these simulations is available online (https://github.com/kdesende/NeuralSignalsOfPerformanceMonitoring, Desender, 2021; copy archived at swh:1:rev:892bf43d65e1adec7a5872f4cb911eb9d28d8633). In the ‘continuation of pre-decisional accumulation’ model, post-decisional accumulation was simply a continuation of precisely the same accumulation process that drove the initial decision. In this model, how the post-decisional DV informs confidence is determined by which bound is reached to trigger the initial decision: if the upper bound is initially reached, then a more positive post-decisional DV translates into higher confidence and a more negative DV to lower confidence; conversely, if the lower bound is initially reached, a more positive DV translates into lower confidence and a more negative DV to higher confidence. For this reason, we flipped the sign of the post-decisional DV on all trials where the initial decision was triggered by the upper bound being reached, ensuring that the DV could be used to inform confidence and error detection judgements in an identical fashion on all trials. The ‘selective error evidence’ model, by contrast, only accumulated the absolute of post-decisional evidence samples that conflicted with the initial choice (negatively/positively signed evidence if the upper/lower bound triggered the initial decision, respectively). In both models, discrete error detection was implemented by putting a single criterion at the 20% quantile; graded confidence was implemented by imposing multiple criteria which divided post-decisional evidence into six equal-sized bins. As can be seen in Appendix 1—figure 1, for both models the relation between confidence and initial choice accuracy and between error detection and initial choice accuracy was highly similar. Next, we examined how well both of these models can explain three key findings in the field of confidence described in the main text. First, it is well known that confidence increases with evidence strength for correct trials but decreases with evidence strength for errors. To simulate this effect, in addition to the trials reported in Figure S1, we simulated an additional 5000 trials with an (increased) drift rate of 2. Second, the relation between confidence and accuracy is known to be stronger when participants take more time for their initial decision. To simulate this, in addition to the trials reported in Figure S1, we simulated an additional 5000 trials with an (increased) decision bound of 3. Third, the relation between confidence and accuracy is known to be stronger when participants are allowed more time following an initial decision to provide their confidence report. To simulate this, in addition to the trials reported in Figure S1, we simulated an additional 5000 trials with (decreased) post-decision accumulation time of 200 ms. As can be seen in Appendix 1—figure 2, both the continuation of pre-decisional accumulation model (upper panel) and the model that selectively accumulates error evidence (lower panel) provide highly similar predictions concerning these three empirical findings.
